# Post-stroke Chorea in the Neurology Department of Ignace Deen Hospital of Conakry, Guinea

**DOI:** 10.7759/cureus.35515

**Published:** 2023-02-27

**Authors:** Idrissa Doumbouya, Souleymane Djigue Barry, Mohamed Lamine Toure, Djénabou Négué Barry, Karinka Diawara, Mohamed Traoré, Mamadou Hady Diallo, Fodé Abass Cissé, Amara Cissé

**Affiliations:** 1 Neurology, Ignace Deen Hospital, Gamal Abdel Nasser University of Conakry, Conakry, GIN

**Keywords:** conakry, middle cerebral artery, basal ganglia, stroke, chorea

## Abstract

Introduction: Chorea is an uncommon complication of stroke. The pathophysiology, the exact location of the lesions, and the evolution of this type of chorea are still poorly understood. The objective was to describe the epidemiological, clinical, and imaging profile of post-stroke chorea in a tropical environment in the context of a stroke epidemic.

Material and Methods: We conducted a five-year retrospective observational study from 2015 to 2020 on stroke patients who presented with chorea in our department. Epidemiological, clinical, and imaging data were registered.

Results: Fourteen patients presented with chorea after their stroke, a frequency of 0.6%. The average age was 57.1 years with a male predominance. Hypertension was the cardiovascular risk factor in half of the patients; three patients (21.4) were diabetic. Chorea was the initial manifestation of the stroke in eight patients (57.1%). Thirteen patients (92.9%) had an ischaemic stroke and one had a cerebral haemorrhage. The middle cerebral artery (MCA) was involved in nine patients (64.3%), the anterior cerebral artery (ACA) in three patients (21.4%), and two patients (14.3%) had posterior cerebral artery (PCA) involvement. The lesions were cortical in five patients (35.7%), five other patients (35.7%) had a deep location, and four patients (28.6%) had both deep and cortical locations of their lesions. The structures affected were the lentiform nucleus (50%), the insula (35.7%), the caudate nucleus (14.3%), and the thalamus (14.3%).

Conclusion: Post-stroke chorea is poorly studied in the tropics. In the presence of any acute abnormal movement associated with cardiovascular risk factors, post-stroke chorea should be considered. Recovery is rapid when treated early.

## Introduction

Abnormal movement after stroke is most commonly associated with damage to the basal ganglia (44%) and thalamus (37%) [1]. The likelihood of developing abnormal movement after infarction of the deep nuclei and thalamus is three times higher than after cortical infarction [2]. Hyperkinetic symptoms such as chorea occur rarely in stroke in the range of 1-4% [3]. Ching et al. report that the incidence of post-stroke hemichorea was 0.54% (27 of 5,009 patients) [4]. According to the Lausanne Stroke Registry, chorea accounts for 38% of all post-stroke abnormal movements [1]. 

Although the precise mechanisms underlying the pathogenesis of these conditions have not been fully characterised, disturbances in the crosstalk between inhibitory and excitatory circuits resulting from vascular damage are proposed as the underlying cause. The gamma-aminobutyric acid (GABA)ergic and dopaminergic systems play a key role in post-stroke abnormal movement [5]. Classically, it is caused by lesions in the subthalamic nuclei; however, it can be associated with lesions in other basal ganglia. Patients with chorea of vascular origin usually have an acute or subacute onset of symptoms contralateral to the lesion [6]. Small vessel infarcts are the most common type of stroke implicated in the occurrence of chorea. Chorea presenting after cardioembolism, large artery infarction, and intracerebral haemorrhage has also been reported [3].

The lack of data on vascular chorea in Africa makes it difficult to assess its impact and outcome in stroke patients. Thus, the aim of this study was to describe the epidemiological, clinical, and imaging characteristics of post-stroke chorea in Conakry, Guinea.

## Materials and methods

This was a retrospective observational study of five years duration from 2015 to 2020, conducted at the neurology department of Ignace Deen Hospital, Conakry, Guinea. We included all patients who developed chorea following a stroke confirmed by brain imaging. Post-stroke chorea has been defined as an involuntary arrhythmic movement, which occurs suddenly, briefly and unrepeatedly following a stroke [6]. Records were accessed from the stroke register of the Neurology Department of Ignace Deen Hospital.

Patients with a family history of chorea, brain tumours, Huntington's disease, infectious, toxic or autoimmune causes, and non-ketotic hyperglycaemia were excluded from the study. For each patient, age, professional category, and known cardiovascular risk factors (hypertension, diabetes, dyslipidemia, HIV, smoking, alcohol, sedentary lifestyle, and oral contraceptives) were investigated. The time to onset of hemichorea following the stroke has been registered, and the severity of the stroke was assessed using the National Institute of Health Stroke Scale (NIHSS) score. CT scans were performed in all patients to confirm the stroke and to investigate the location (cortical or deep). The Alberta Stroke Program Early CT (ASPECT) score was used to describe middle cerebral artery ischaemic involvement [7]. ECG was performed in all patients and abnormalities were noted. For patients with ischemic stroke, echocardiographic and neck vessel abnormalities were collected. 

Etiologically, cerebral infarcts were classified according to the trial of ORG 10172 in acute stroke treatment (TOAST) [8]. Angioscan was performed to detect vascular malformation. Treatment of chorea was recorded; the evolution was studied according to whether the hemichorea improved or was resistant to treatment. 

Data analysis was done by Epi Info™ 7.1, a database and statistics program for public health professionals (CDC, Atlanta, Georgia, United States). Quantitative variables were expressed as mean and the qualitative variables were expressed as frequency.

## Results

During the study period, 2500 patients were followed up for stroke in the department. Of these, 14 developed their hemichorea i.e. 0.6% of patients; there were five females (35.7%) and nine males (64.3%) with a sex ratio of 1.8. The average age was 57.1 ± 10.6 years (extremes: 33 and 73 years). Half of the patients had hypertension and three patients (21.4%) had diabetes (Table 1).

**Table 1:** Demographics characteristics of patients

Variables	Number (n=14)	Percentage (%)
Sex		
Male	9	64.3
Female	5	35.7
Sex ratio M/F: 1.8		
Age		
Average age 57.1 ± 10.6 (extremes: 33 and 73 years)		
Age groups		
33 - <43	1	7.1
43 - <53	4	28.6
53 - <63	5	35.7
63 - <73	3	21.4
≥73	1	7.1
Cardiovascular risk factors		
Hypertension	7	50
Diabetes	3	21.3
Smoking	2	14.3
Sedentary lifestyle	2	14.3
Dyslipidemia	2	14.3
Oral contraceptives	1	7.1
Alcohol	1	7.1

Chorea was unilateral in 13 patients (92.1%); it was the initial manifestation of the stroke in eight patients (57.1%) (Video 1), two patients (14.3%) presented their chorea within the first 24 hours, three patients (21.4%) between the second and fifth day and one patient (7.1%) beyond one month. The mean NIHSS of the patients was four, hemiparesis and hemicorporal hypotonia were the associated signs encountered in 78.6% and 71.4% respectively (Table 2).

**Video 1:** Post-stroke left hemichorea discovered on awakening

**Table 2:** Clinical characteristics NIHSS: National Institute of Health Stroke Scale

Variables	Numbers (n=14)	Percentages(%)
Time to onset		
Initial event	8	47.1
First day	2	14.3
2-5 days	3	21.4
More than 5 days	1	7.1
NIHSS Mean 4 ±1		
Associated neurological signs		
Hemiplegia	11	78.6
Hemicorporeal hypotonia	10	71.4
Aphasia	4	28.6
Sensory disorder	2	14.3
Spastic hypertonia	1	7.1
Visual disorder	1	7.1

Thirteen patients (92.9%) had an ischemic stroke and only one patient (7.1%) had a haemorrhagic stroke. The lesions were cortical in five patients (35.7%), another five patients (35.7%) had a deep location, and four patients (28.6%) had both deep and cortical locations of their lesions. There was involvement of the middle cerebral artery (MCA) territory in nine patients (64.3%), involvement of the anterior cerebral artery (ACA) in three patients (21.4%), and involvement of the posterior cerebral artery (PCA) in two patients (14.3%). The M3, M4, and M5 segments of the MCA were affected in 28.6%, 42.9% and 50%, respectively. Among the deep structures, the lentiform nucleus was the most affected (50% of cases), followed by the insular ribbon (35.7%), the caudate nucleus (14.3%), and the thalamus (14.3%) (Table 3). Atherosclerosis was the aetiology found in four patients (28.6%); atrial fibrillation (7,1%) and intra-cavity thrombus ( 7,1%) were the cardiopathies found. Two patients (14.3%) had cerebral lacunas due to hypertension. The cause was not found in six patients (42.9%). The medicines used were haloperidol in 11 patients (78.6%) and tiapride in three patients (21.4%). Twelve patients (85.7 %) had a complete resolution of the chorea and in two patients (14.3 %), the abnormal movement persisted despite the treatment.

**Table 3:** Imaging characteristics MCA: middle cerebral artery; ASPECTS: Alberta Stroke Program Early CT score; PCA: posterior cerebral artery; ACA: anterior cerebral artery

Variables	Number (n=14)	Percentages (%)
Stroke type		
Hemorrhagic	1	7.1
Ischaemic	13	92.9
Arterial territories		
Cortical	5	35.7
Cortical and deep	4	28.6
Deep	5	35.7
Affected arteries		
MCA (ASPECTS)	9	64.3
M1	2	14.3
M2	3	21.4
M3	4	28.6
M4	6	42.3
M5	7	50
M6	3	21.4
Lenticular nucleus	7	50
Insular ribbon	5	35.7
Caudate nucleus	2	14.3
PCA (ASPECTS)	2	14.3
Lobe occipital	1	7.1
Thalamus	2	14.3
ACA	3	21.4
Anterior choroidal artery	1	7.1

## Discussion

Sudden hyperkinetic movements such as hemichorea are a rare presentation of stroke [9]. They are observed either in the acute or chronic phase of a stroke. We report the first documented cases of post-stroke chorea in Guinea. The lack of magnetic resonance imaging (MRI) and the absence of recanalisation therapy were the limitations and difficulties of this study. 

The prevalence of post-stroke chorea is difficult to determine. Alcaron et al. reported on 56 patients with abnormal movements after stroke of which 20 patients (35%) had chorea of vascular origin [10]. Ghika-Schmid et al. reported that chorea was the most common abnormal movement after stroke, representing 0.4-0.54% of cases [1]. The average age (57.1 years) found in our study is lower than that of Parées et al. in a series of 15 patients (73 years) [11]. Carbayo et al., in a series of four patients in 2020, found an average age of 72 years [12]. The lower life expectancy and the early onset of stroke in Guinea would explain the fact that post-stroke chorea victims in Guinea are younger than those reported in the international literature.

Ischaemia was the most common type of stroke in this series, 13/14 patients (92.9%). The MCA was the most affected artery (64.3%); the lentiform nucleus was the most affected basal ganglion (50%). The insular ribbon was involved in 35.7% and cortical lesions were predominant in the MCA territory (64.3%). In the series by Carbayo et al., all patients had an ischemic stroke [12]. Twelve patients (80%) had an ischaemic stroke and three patients (20%) had an intracerebral haemorrhage stroke in the series by Parées et al. [11]. Ghika-Schmid et al. reported that this abnormal movement is typically associated with lesions of the basal ganglia and adjacent white matter in the MCA or PCA territory [1]. Frequently, lesions may be located in the thalamus and lentiform nucleus [10] and less often in the subthalamic nucleus (STN) [4].

Hemichorea is commonly seen with lesions involving the lenticular nucleus or thalamus but has also been reported with lesions involving the subthalamus, stratum, the posterior arm of the internal capsule, radial corona, frontal lobe, parietal lobe, temporal cortex, external capsule, and pons [9]. Cortical lesions were also detected in six patients in the study by Etgen and al. [13], these lesions were located in the territory of the MCA. The mechanism by which cortical lesions lead to hemichorea is not well understood. One hypothesis is that hypoperfusion of the basal ganglia without obvious lesions on MRI could be the underlying cause, but several studies have shown that cortical infarction alone is sufficient to cause hemichorea [11,14]. In this pathway, the subthalamic (excitatory) nucleus is directly activated by cortical inputs, enhancing the inhibitory activity of the medial globus pallidus [15]. Thus, a decrease in cortical signal output due to cortical infarction can induce choreic movements [16]. 

Haloperidol and tiapride were the medications used. Twelve patients (85.7%) had a complete improvement in movement. An observational study in 2010 of 15 patients with post-stroke hemichorea found that eight patients (53%) required no pharmacological treatment due to rapid resolution, while the remaining seven required pharmacological treatment to control the hemichorea. However, most patients had resolution of their symptoms within two months [11]. In cases where choreic movement is persistent or disabling, medical therapy with anti-dopaminergics (haloperidol) may be considered [17]. Atypical neuroleptics such as olanzapine, quetiapine, and sulpiride act by blocking D3 and D4 dopamine receptors and are less likely to cause drug-induced parkinsonism and tardive dyskinesia [16,18].

## Conclusions

Chorea is a rare complication of stroke and remains poorly studied in the tropics. It may occur immediately after the stroke or may be delayed. In the face of any acute abnormal movement associated with cardiovascular risk factors, the diagnosis of post-stroke chorea should be considered and treated. Lesions of the lentiform nucleus and cortex are more associated with hemichorea. The complexity of the cortico-basal motor network explains the deep and cortical location of the vascular lesions. The evolution is most often favourable when treated early.
